# Challenges of diagnostic exome sequencing in an inbred founder population

**DOI:** 10.1002/mgg3.7

**Published:** 2013-04-22

**Authors:** Dimitar N Azmanov, Teodora Chamova, Rick Tankard, Vladimir Gelev, Michael Bynevelt, Laura Florez, Dochka Tzoneva, Dora Zlatareva, Velina Guergueltcheva, Melanie Bahlo, Ivailo Tournev, Luba Kalaydjieva

**Affiliations:** 1 Laboratory for Molecular Genetics, Centre for Medical Research/Western Australian Institute for Medical Research, The University of Western AustraliaPerth, WA, Australia; 2 Department of Neurology, Medical UniversitySofia, Bulgaria; 3 Bioinformatics Division, The Walter and Eliza Hall InstituteMelbourne, VIC, Australia; 4 Faculty of Chemistry and Pharmacy, Sofia UniversitySofia, Bulgaria; 5 Department of Surgery, School of Medicine, The University of Western AustraliaPerth, WA, Australia; 6 Neurological Intervention and Imaging Service (WA), Sir Charles Gairdner HospitalPerth, WA, Australia; 7 Department of Anesthesiology and Intensive Care, University Hospital “Alexandrovska”Sofia, Bulgaria; 8 Department of Diagnostic Imaging, University Hospital “Alexandrovska”Sofia, Bulgaria; 9 Department of Mathematics and Statistics, The University of MelbourneMelbourne, VIC, Australia; 10 Department of Cognitive Science and Psychology, New Bulgarian UniversitySofia, Bulgaria

**Keywords:** Diagnostic exome sequencing, dysequilibrium syndrome, founder mutations, Roma/Gypsies, VLDLR

## Abstract

Exome sequencing was used as a diagnostic tool in a Roma/Gypsy family with three subjects (one deceased) affected by lissencephaly with cerebellar hypoplasia (LCH), a clinically and genetically heterogeneous diagnostic category. Data analysis identified high levels of unreported inbreeding, with multiple rare/novel “deleterious” variants occurring in the homozygous state in the affected individuals. Step-wise filtering was facilitated by the inclusion of parental samples in the analysis and the availability of ethnically matched control exome data. We identified a novel mutation, p.Asp487Tyr, in the *VLDLR* gene involved in the Reelin developmental pathway and associated with a rare form of LCH, the Dysequilibrium Syndrome. p.Asp487Tyr is the third reported missense mutation in this gene and the first example of a change affecting directly the functionally crucial β-propeller domain. An unexpected additional finding was a second unique mutation (p.Asn494His) with high scores of predicted pathogenicity in *KCNV2*, a gene implicated in a rare eye disorder, retinal cone dystrophy type 3B. This result raised diagnostic and counseling challenges that could be resolved through mutation screening of a large panel of healthy population controls. The strategy and findings of this study may inform the search for new disease mutations in the largest European genetic isolate.

A Roma/Gypsy family with three subjects (one deceased) (Fig. S1) affected by a defect in brain development was referred for diagnostic investigations. The clinical features (Table S1) included global developmental delay, moderate to severe intellectual deficit, nonprogressive severe truncal ataxia, dysarthric speech, gaze-evoked nystagmus, mild intentional tremor, and pyramidal signs. Neuroimaging (Fig. S2) showed global small brain, pontocerebellar hypoplasia, and mild to moderate cortical thickening with gyral simplification more pronounced in the frontal and temporal regions. The phenotype was classified broadly as lissencephaly with cerebellar hypoplasia (LCH), a heterogeneous diagnostic category of cortical malformations where some patients have defects in the Reelin neuronal migration pathway but a significant proportion of cases remain unexplained (reviewed in Ross et al. 2001; Barkovich 2012). LCH genetic heterogeneity prompted us to choose exome sequencing as an efficient diagnostic approach. The analysis included the two living patients and one set of parents (Fig. S1). Written informed consent was obtained from the parents; the study complies with the ethical guidelines of the institutions involved.

Exome capture (Illumina TruSeq) and sequencing (Illumina HiSeq 2000, Illumina Inc., San Diego, CA) were performed by Axeq Technologies (Seoul, South Korea). After initial quality control, data analysis included alignment to the hg19 reference genome (Li and Durbin 2009), variant calling in SAMtools (Li et al. 2009) using default parameters, and identification of variants in dbSNP135 (http://www.ncbi.nlm.nih.gov/projects/SNP/). Variants were annotated using ANNOVAR (Wang et al. 2010) version 23 October 2012, and ANNOVAR-formatted databases based on the UCSC Known Gene (“hg19_knownGene”), the 1000 Genomes project (http://www.1000 genomes.org/) (“hg19_ALL.sites.2012_02”), and the NHLBI Exome Sequencing Project (http://evs.gs.washington.edu/EVS/; “hg19_esp6500_all”). From the exome sequencing data, a set of 5521 polymorphic markers (intermarker distance ≥0.15 cM, in approximate linkage equilibrium, average heterozygosity 0.42) were extracted (Smith et al. 2011) and used to estimate inbreeding coefficients (Leutenegger et al. 2003). The search for the disease-causing mutation focused on rare variants (<1% in public databases) including nonsense, exonic indels, affecting splicing sites, and missense variants predicted to be deleterious by Polyphen2 (Adzhubei et al. 2010) (ANNOVAR database “hg19_ljb_pph2”) and SIFT (Ng and Henikoff 2001) (ANNOVAR database “hg19_avsift”).

In contrast to the reported genealogy, inbreeding analysis revealed close parental consanguinity (Fig. S1) which, together with the pedigree structure suggesting autosomal recessive inheritance, led us to assume autozygosity for a rare/unique deleterious variant. Out of a total of 63,000–68,000 variants present in each affected subject, our step-wise filtering strategy (Fig. S3) identified ca. 500 rare “deleterious” changes (0.73% of all variants) that were homozygous in each patient, including 309 shared by both patients. The final filtering criteria required heterozygosity in the parents and no homozygosity among control Roma exomes. This left two novel missense mutations in neighboring genes on chromosome 9p24: a G>T (hg19 chr9:2645720; RefSeq NM_003383.3, exon10: c.1459G>T; NP_003374.3: p.(Asp487Tyr)) in *VLDLR* and an A>C (hg19 chr9: 2729569; RefSeq NM_133497.3, exon2: c.1480A>C; NP_598004.1: p.(Asn494His)) in *KCNV2* (Fig. 1a). The predicted amino acid substitutions were nonconservative: from the acidic polar Aspartic acid to the aromatic nonpolar Tyrosine in VLDLR and from the neutral polar Asparagine to the basic polar Histidine in KCNV2. Both affected evolutionary conserved positions, with deleterious effects predicted with very high probability by PolyPhen-2 and SIFT (Fig. 1b and c). Both genes have been implicated in rare Mendelian disorders: *VLDLR* (very low-density lipoprotein receptor) – cerebellar ataxia, mental retardation, and disequilibrium syndrome 1, CAMRQ1, MIM#224050 and *KCNV2* (voltage-gated potassium channel subunit Kv8.2) – retinal cone dystrophy, RCD3B, MIM#610356.

**Figure 1:**
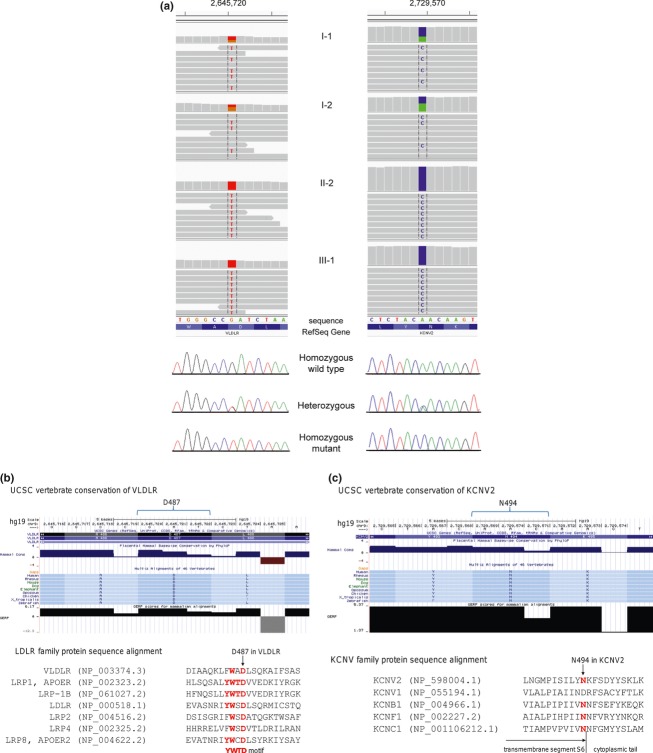
Two unique missense variants identified by exome sequencing in the affected family. (a) Integrative Genomics Viewer snapshot of the short reads alignment from the exome sequencing (upper panel) and confirmatory Sanger sequencing (lower panel); left G>T (hg19 chr9:2645720) in *VLDLR*, right A>C (hg19 chr9:2729569) in *KCNV2*. (B, C) UCSC and Multalign (Corpet 1988) analysis of the evolutionary conservation of VLDLR Asp487 (b) and KCNV2 Asn494 (c) interspecies (upper panel) and protein family members (lower panel) comparisons. The VLDLR mutation affects a strictly conserved amino acid residue in the consensus repeat motif of the second blade in the β-propeller structure of the protein. The deleteriousness prediction scores in PolyPhen-2 equaled 1.00 for both mutations; SIFT scores were 0.00 for the VLDLR and 0.05 for the KCNV2 change. VLDLR, very low-density lipoprotein receptor; UCSC, University of California, Santa Cruz, genome browser; SIFT, sorting intolerant from tolerant algorithm.

The p.Asp487Tyr mutation in *VLDLR* could explain the neurological phenotype, classifying the affected individuals as VLDLR-associated Dysequilibrium Syndrome (DES), a rare condition with eight disease-causing (two missense) mutations reported to-date (Boycott et al. 2009; Kolb et al. 2010; Ali et al. 2012). The VLDL receptor is part of the Reelin developmental pathway, orchestrating the migration of glutamatergic neurons into cortical layers, the alignment of pyramidal neurons in the hippocampus, and the dispersal of Purkinje cells in the cerebellum (D'Arcangelo et al. 1995, 1999; Trommsdorff et al. 1999). Reelin signaling is regulated through internalization and rapid uncoupling of the ligand from the VLDL receptor due to conformational changes at endosomal pH, whereupon the ligand is targeted for lysosomal degradation and the receptor is recycled to the cell membrane (Fig. 2) (Duit et al. 2010; Reddy et al. 2011). The p.Asp487Tyr mutation can be predicted to disrupt the β-propeller protein domain, shown to be essential for ligand release and receptor recycling in the closely related LDL receptor (Rudenko et al. 2002; Beglova and Blacklow 2005). The pathogenic effect may involve protein misfolding and impaired trafficking, as proposed for another *VLDLR* mutation, p.Asp521His (Boycott et al. 2009) or, alternatively, may interfere with ligand dissociation upon internalization (Fig. 2).

**Figure 2:**
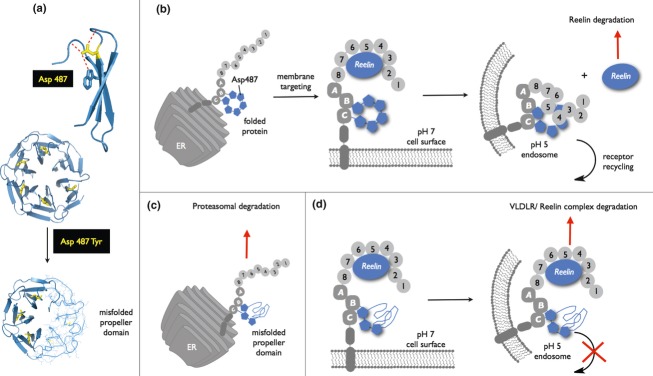
Mechanisms of action of the very low-density lipoprotein receptor (VLDL) receptor and potential pathogenic effects of the p.Asp487Tyr substitution. (a) In the consensus repeat motifs (Tyr-Trp-Thr-Asp), Asp residues serve as clasps between adjacent blades of the β-propeller, stabilizing the structure by hydrogen-bonding with the backbone and Trp side chains (red dotted lines). The mutation is predicted to disrupt these interactions, leading to a misfolded β-propeller. (b) The correctly folded receptor protein is targeted to the neuronal surface. Upon Reelin binding the complex is internalized, with conformational changes induced by the endosomal pH leading to dissociation and lysosomal degradation of the ligand, while the receptor is recycled to the cell membrane. (c) The misfolded mutant receptor may be retained in the endoplasmic reticulum and targeted for degradation. (d) Alternatively, correct membrane targeting and ligand binding are followed by lack of conformational changes at acid pH, impaired ligand release, and targeting the entire ligand-receptor complex for degradation. The ribbon diagram in (a) was constructed in PyMol (http://www.pymol.org) from the corresponding crystal structure of the YWTD repeat of the LDL receptor (PDB ID 1IJQ). Schematic in (b-d) adapted from Beglova et al. (2005).

In contrast to the *VLDLR* mutation, which was an obvious candidate accounting for the brain malformation and ensuing phenotype, the *KCNV2* change was an unexpected finding of unknown clinical significance and counseling implications. Retinal cone dystrophy type 3B is a slowly progressing disorder of variable severity, whose diagnosis relies on specific electroretinographic findings (Robson et al. 2010). The sustained cooperation required during electroretinography was unachievable in our patients in view of their mental retardation, and no relevant information could be obtained from the care providers, leading us to resort to mutation screening in ethnically matched controls as the feasible approach to plausibility assessment. A panel of healthy Roma controls from a range of subisolates (Kalaydjieva et al. 2005) was tested using custom-designed TaqMan® SNP Genotyping Assays (Applied Biosystems, Mulgrave, VIC, Australia) (Table S2). The *VLDLR* p.Asp487Tyr variant was not detected in 566 control subjects, suggesting that it is a private mutation confined to this consanguineous family. By contrast, the *KCNV2* variant was very common across subisolates, with 101 carriers (14%) and 8 homozygotes (1.1%) identified among 721 controls. This unusually high frequency, with an improbable prevalence of ∼1/100 of presumably affected individuals (under the assumption of complete penetrance) indicated that, contrary to bioinformatics predictions, the *KCNV2* change was a polymorphism, not a pathogenic mutation.

The population genetic characteristics of the Roma population, with strong founder effects, genetic drift, and limited diversity, have been described in previous studies (reviewed in Kalaydjieva et al. 2005). What has become apparent from recent exome sequencing data is a surprisingly high level of inbreeding (this study and Guergueltcheva et al. 2012) that could be due to unrecognized consanguinity and the cumulative effects of historical endogamy and small population size. As a result, Roma exomes present with a large absolute number and proportion of all high quality exome variants per individual of homozygous “deleterious” variants, significantly in excess of the proportion observed in outbred Caucasian samples available in-house (one sided *t*-test, unequal variance, *P* = 4e^−6^, df = 4.548) (Table S3). The findings emphasize the need for custom-designed family- and population-based approaches to diagnostic exome sequencing in inbred founder populations. In our study, filtering out the plethora of “candidate mutations” was made possible by the inclusion of parental data and comparison to other Roma exomes. The additional challenge of a second unique “pathogenic” mutation, not found in over 6500 exomes in public databases, would have remained unresolved if population-specific data were unobtainable or ambiguous, highlighting the medical and ethical dilemmas in this type of analyses and the need for ethnically matched controls, as well as for further improvement of bioinformatics predictions.

## Supporting Information

**Figure S1.** Pedigree affected by lissencephaly with cerebellar hypoplasia. The family belonged to a young, strictly endogamous Roma/Gypsy subisolate structured into multiple small clans (Kalaydjieva et al. 2005). The family reported a single consanguineous marriage at 3rd cousin level, between II-3 and II-4 (not shown), in contrast to the high inbreeding coefficients estimated using FEstim and SNPs extracted from the exome sequencing data: 0.038 (I-1), 0.062 (I-2), 0.05 (II-2), and 0.057 (III-1). Exome sequencing identified two unique variants, in *VLDLR* and *KCNV2*, that satisfied all filtering criteria.

**Figure S2.** Brain MRI (magnetic resonance imaging) of subject II-2. (A) Sagittal T1W and (B) Coronal T1 Inversion recovery demonstrating marked cerebellar hypoplasia, particularly affecting the inferior hemispheres and vermis as a whole. The pons is also notably small. (C) Axial T2W showing mild to moderate cortical thickening (pachygyria) with simplification of the gyral architectural folding.

**Figure S3.** Step-wise filtering of the variants identified by exome sequencing in the affected individuals. The search for the disease mutation was based on the assumption of a rare/unique variant homozygous in both patients, heterozygous in the parents, and not homozygous in 25 Roma exomes available in-house. *Primary quality control retained for further analysis variants with quality scores ≥20 and coverage ≥4×, excluding changes located in segmental duplications and simple repeats; **∧**Variants defined as deleterious included nonsynonymous amino acid substitutions with a Polyphen2 score >0.8 and SIFT score ≤0.05, splice-site (±15 nucleotides), nonsense and nonstop changes, as well as small in-frame or frameshift insertion/deletions.

**Table S1.** Clinical and neuroimaging findings in the affected subjects.

**Table S2.** Primers, PCR conditions, and TaqMan® probes used in the analysis of *VLDLR* c.1459G>T and *KCNV2* c.1480A>C. (A) Primers and PCR conditions for fragment amplification and Sanger sequencing. (B) TaqMan ® assay primers and probes used in the population screening.

**Table S3.** (A) Proportion of homozygous “deleterious” variants relative to all high-quality variants observed in individuals from the Roma family studied and in 28 outbred exomes after filtering. (B) Boxplot of homozygous “deleterious” variants relative to all high-quality variants observed in individuals from the Roma family studied and in 28 outbred exomes after filtering. The circle represents an outlier, as determined by the “boxplot” function in the statistical software R (http://www.r-project.org/).
