# Supplementary material for: Challenges of diagnostic exome sequencing in an inbred founder population
Source: Mol Genet Genomic Med. 2013 Apr 22;1(2):71–6. doi: 10.1002/mgg3.7 (PMC3865571; doi:10.1002/mgg3.7)

**Supp. Figure S1.** Pedigree affected by lissencephaly with cerebellar hypoplasia. The family belonged to a young, strictly endogamous Roma/Gypsy sub-isolate structured into multiple small clans [Kalaydjieva et al., 2005]. The family reported a single consanguineous marriage at 3^rd^ cousin level, between II-3 and II-4 (not shown), in contrast to the high inbreeding coefficients estimated using FEstim and SNPs extracted from the exome sequencing data: 0.038 (I-1), 0.062 (I-2), 0.05 (II-2) and 0.057 (III-1). Exome sequencing identified two unique variants, in *VLDLR* and *KCNV2*, that satisfied all filtering criteria.


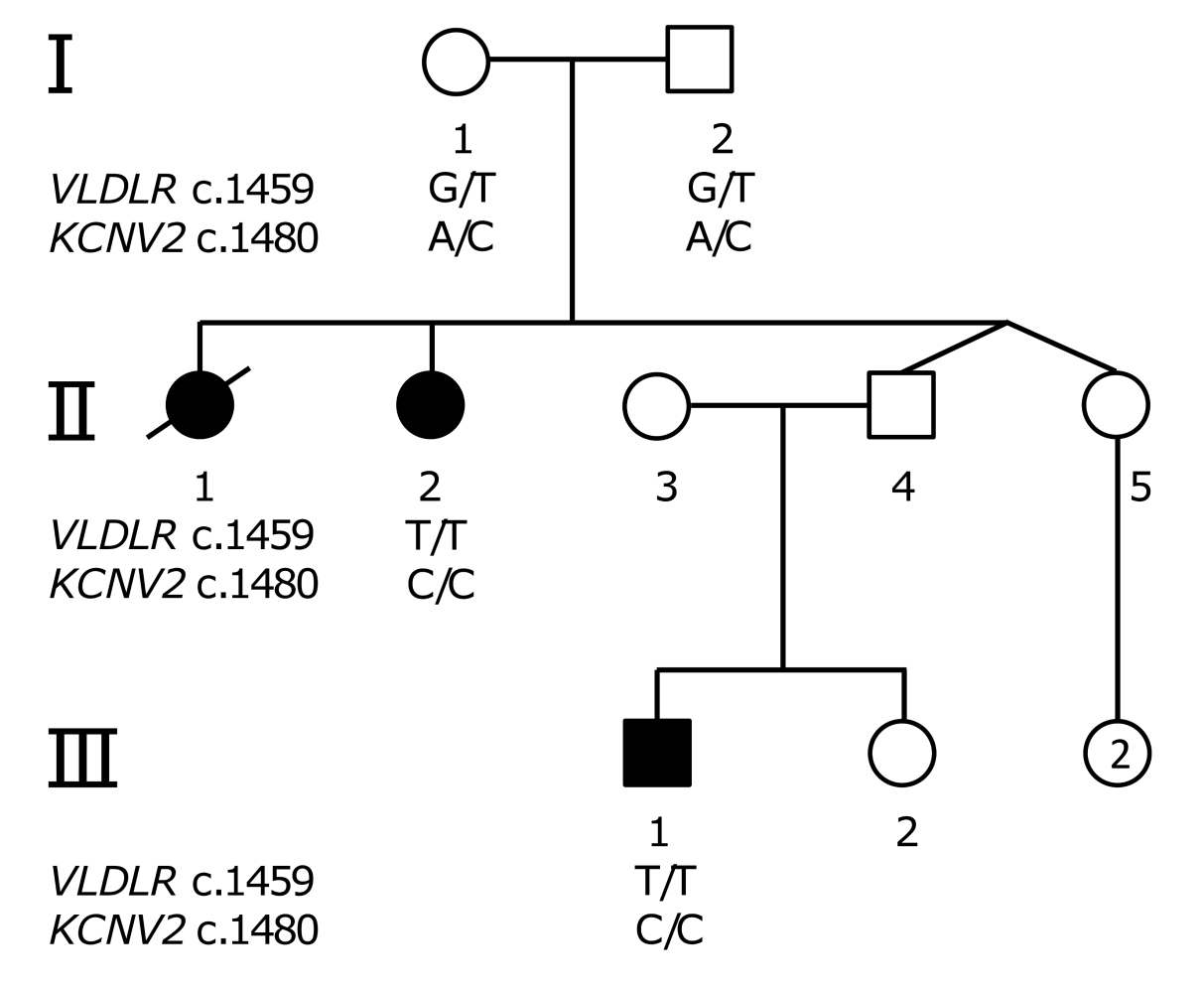


**Supp. Figure S2.** Brain MRI (magnetic resonance imaging) of subject II-2. **A:** Sagittal T1W and **B:** Coronal T1 Inversion recovery demonstrating marked cerebellar hypoplasia, particularly affecting the inferior hemispheres and vermis as a whole. The pons is also notably small. **C:** Axial T2W showing mild to moderate cortical thickening (pachygyria) with simplification of the gyral architectural folding.


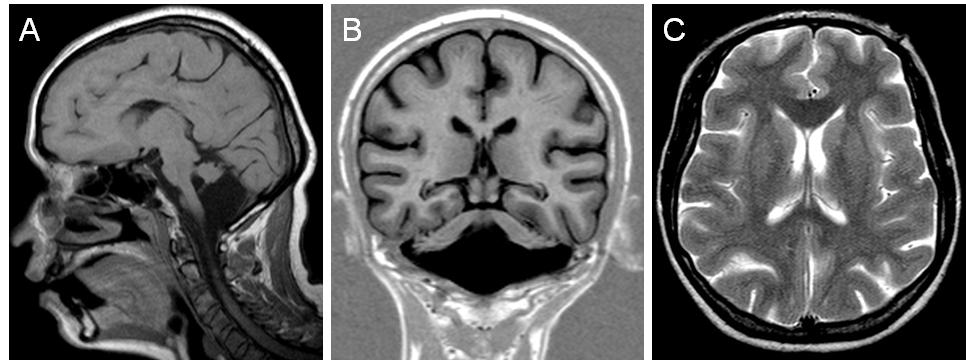


**Supp. Figure S3**. Step-wise filtering of the variants identified by exome sequencing in the affected individuals. The search for the disease mutation was based on the assumption of a rare/unique variant homozygous in both patients, heterozygous in the parents and not homozygous in 25 Roma exomes available in-house. *****Primary quality control retained for further analysis variants with quality scores ≥20 and coverage≥4X, excluding changes located in segmental duplications and simple repeats; **^**Variants defined as deleterious included non-synonymous amino acid substitutions with a Polyphen2 score >0.8 and SIFT score ≤ 0.05, splice-site (±15 nucleotides), nonsense and non-stop changes, as well as small in-frame or frameshift insertion/deletions.


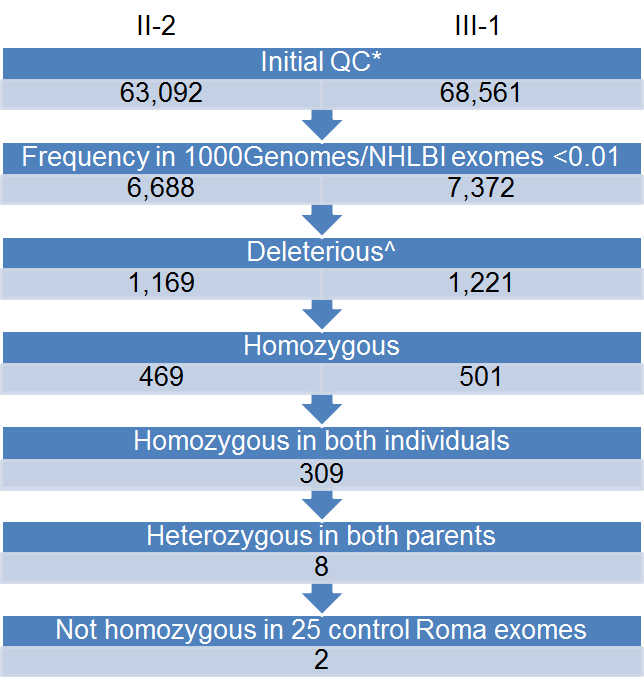


**Supp. Table S1.** Clinical and neuroimaging findings in the affected subjects.

| **Patient** | **II-1** | **II-2** | **III-1** |
| --- | --- | --- | --- |
| **Year of birth** | 1969 | 1971 | 1981 |
| **Sex** | F | F | M |
| **Developmental milestones (y):** |  |  |  |
| **Walking with support** | 11-12 | 10 | 10 |
| **Talking – simple utterances** | 16 | 16 | 10 |
| **History of seizures** | No | No | No |
| **Most recent examination (year)** | 1997* | 2011 | 2011 |
| **Height (cm)** | “short” | 150 | 154 |
| **Weight (kg)** | ND | 51 | 52 |
| **Head circumference (cm)** | ND | 50 | 45 |
| **High-arched palate** | Yes | Yes | Yes |
| **Hypertelorism** | No | No | Yes |
| **Strabismus** | Yes | Yes | No |
| **Neurological manifestations** |  |  |  |
| **Muscle hypotonia** | Yes | Yes | Yes |
| **Ataxia – SARA scores^#^** |  |  |  |
| **Gait (0-8)** | Severe | 7 | 8 |
| **Stance (0-6)** | Severe | 6 | 6 |
| **Sitting (0-6)** | Mild | 1 | 1 |
| **Speech disturbance (0-6)** | Yes | 4 | 4 |
| **Nose-finger test (R+L/2) (0-4)** | Mild | 1 | 1 |
| **Gaze-evoked horizontal nystagmus** | Yes | Yes | Yes |
| **Tendon reflexes** | Brisk | Brisk | Brisk |
| **Plantar responses** | No | No | No |
| **Intellectual deficit** | Moderate | Moderate | Severe |
| **Brain imaging** | No data | MRI | CT scan |
| **Global small brain** |  | Yes | Yes |
| **Cerebral cortex** |  | Fronto-temporal pachygyria, gyral simplification | Cortical thickening |
| **Cerebellar hypoplasia** |  | Marked generalized | Marked generalized |
| **Brainstem** |  | Small | Small |

^*^Patient II-1 was examined during a home visit. ^#^SARA (Scale for the Assessment and Rating of Ataxia [Schmitz-Hübsch et al., 2006]) scores in the 1^st^ column indicate increasing severity, 0 signifies lack of impairment. Some SARA tests (e.g. finger chase) could not be performed due to the patients’ intellectual deficit.

Schmitz-Hübsch T, du Montcel ST, Baliko L, Berciano J, Boesch S, Depondt C, Giunti P, Globas C, Infante J, Kang JS, Kremer B, Mariotti C et al. 2006. Scale for the assessment and rating of ataxia: development of a new clinical scale. Neurology 66:1717-1720.

**Supp. Table S2.** Primers, PCR conditions and TaqMan® probes used in the analysis of *VLDLR* c.1459G>T and *KCNV2* c.1480A>C

**A:** Primers and PCR conditions for fragment amplification and Sanger sequencing

| Amplicon including | *VLDLR* c.1459G>T | *KCNV2* c.1480A>C |
| --- | --- | --- |
| Forward primer | AGGCAAAGAGCCAAGTCTGA | CAATTCCATCCTGCTTTCCT |
| Reverse primer | CCCCAGGTAATTCGATGCTA | TTCTTTCTGGCTCTCTGCAT |
| Product size bp | 297 | 254 |
| Taq polymerase | Kapa ReadyMix* | Kapa ReadyMix* |
| DMSO | 5% | 5% |
| Initial denaturing | 95°C/ 2 min | 95°C/ 2 min |
| Denaturing | 94°C/ 30 sec | 94°C/ 20 sec |
| Annealing (touch down -0.5°C/cycle) | 63-58°C/ 30 sec | 60-55°C/ 15 sec |
| Extension | 72°C/ 30 sec | 72°C/ 60 sec |
| Total number cycles | 30 | 40 |
| Final extension | 72°C/ 2 min | 72°C/ 7 min |

*KapaBiosystems catalogue # KK1006 (Thebarton, SA, Australia)

**B:** TaqMan ® assay primers and probes used in the population screening

| Amplicon including | *VLDLR* c.1459G>T | *KCNV2* c.1480A>C |
| --- | --- | --- |
| Forward primer | TGACATTGCTGCCCAGAAACTATT | GCATTGCTTTTGGGATCATTCTCAA |
| Reverse primer | GACACCACAAAAGGAACTGAAAGT | CCCTCTCCCTGCGTATGG |
| FAM-labelled probe | TGGGCCTATCTAAG | CCATCCTCTACCACAAGT |
| VIC-labelled probe | TGGGCCGATCTAAG | CCATCCTCTACAACAAGT |

**Supp. Table S3.** **A:** Proportion of homozygous “deleterious” variants relative to all high quality variants observed in individuals from the Roma family studied and in 28 outbred exomes after filtering.

|  | Individual exomes from the Roma family | Exomes from outbred controls |
| --- | --- | --- |
| Mean | 0.0072 | 0.0049 |
| Median | 0.0073 | 0.0049 |
| Range | 0.0070–0.0074 | 0.0045–0.0056 |

**B:** Boxplot of homozygous “deleterious” variants relative to all high quality variants observed in individuals from the Roma family studied and in 28 outbred exomes after filtering. The circle represents an outlier, as determined by the 'boxplot' function in the statistical software R (<http://www.r-project.org/>).


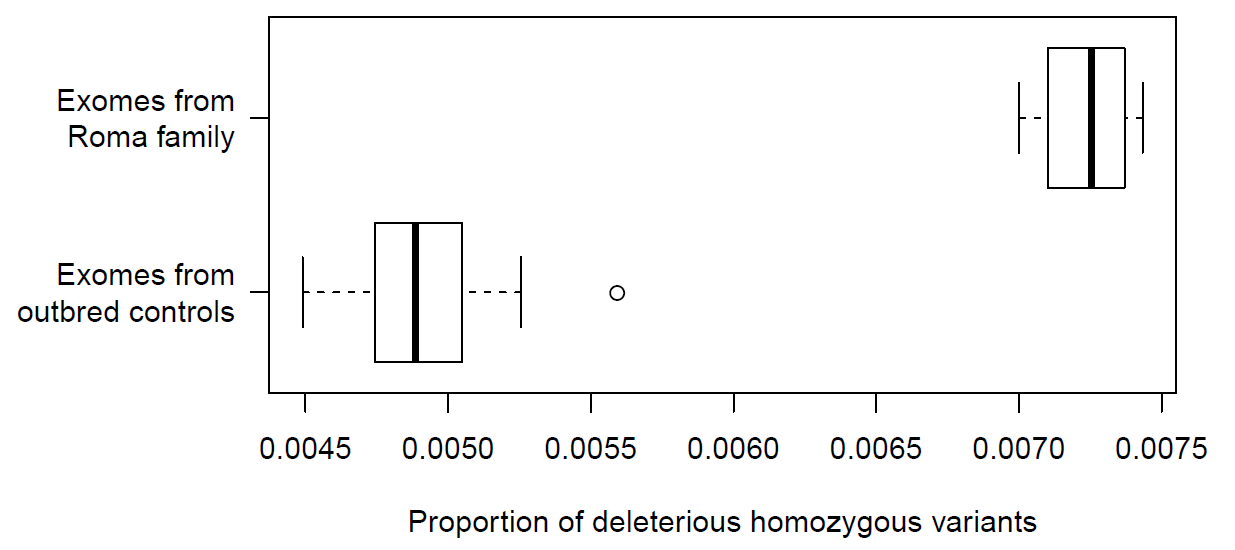

Supplement: Supplementary file 1 [file mgg30001-0071-SD1.doc]
